# Getting to Know Endometriosis-Related Infertility Better: A Review on How Endometriosis Affects Oocyte Quality and Embryo Development

**DOI:** 10.3390/biomedicines9030273

**Published:** 2021-03-09

**Authors:** Mara Simopoulou, Anna Rapani, Sokratis Grigoriadis, Agni Pantou, Petroula Tsioulou, Evangelos Maziotis, Despina Tzanakaki, Olga Triantafyllidou, Theodoros Kalampokas, Charalampos Siristatidis, Panagiotis Bakas, Nikolaos Vlahos

**Affiliations:** 1 Laboratory of Physiology, Medical School, National and Kapodistrian University of Athens, 75, Mikras Asias, 11527 Athens, Greece; rapanianna@gmail.com (A.R.); sokratis-grigoriadis@hotmail.com (S.G.); agni.pantos@gmail.com (A.P.); petroulatsi@yahoo.gr (P.T.); vagmaziotis@gmail.com (E.M.); 2 Assisted Reproduction Unit, Second Department of Obstetrics and Gynecology, Aretaieion Hospital, Medical School, National and Kapodistrian University of Athens, 76, Vasilisis Sofias Avenue, 11528 Athens, Greece; dtzanakaki@gmail.com (D.T.); triantafyllidouolga@gmail.com (O.T.); kalamp@yahoo.com (T.K.); harrysiris@gmail.com (C.S.); p_bakas@yahoo.com (P.B.); nfvlahos@gmail.com (N.V.); 3 Centre for Human Reproduction, Genesis Athens Clinic, 14-16, Papanikoli, 15232 Athens, Greece

**Keywords:** endometriosis, infertility, oocyte, embryo, quality

## Abstract

Endometriosis-related infertility describes a case of deteriorated fecundity when endometriosis is diagnosed. Numerous mechanisms have been proposed in an effort to delineate the multifaceted pathophysiology that induces impairment of reproductive dynamics in patients with endometriosis. In this critical analysis, authors present the plethora of molecular events that are entailed and elaborate on how they potentially impair the oocyte’s and embryo’s competence in patients with endometriosis. Reactive oxygen species, dysregulation of the immune system and cellular architectural disruption constitute the crucial mechanisms that detrimentally affect oocyte and embryo developmental potential. The molecular level impairment of the reproductive tissue is discussed, since differentiation, proliferation and apoptosis constitute focal regulatory cellular functions that appear severely compromised in cases of endometriosis. Mapping the precise molecular mechanisms entailed in endometriosis-related infertility may help delineate the complex nature of the disorder and bring us a step closer to a more personalized approach in understanding, diagnosing and managing endometriosis-related infertility.

## 1. Introduction

Endometriosis constitutes one of the numerous medical enigmas resulting in compromised fertility. It is a chronic benign disease characterized by chronic inflammation, principally affecting women of reproductive age. The vague clinical symptoms regularly accompanying endometriosis render a timely and precise diagnosis rather challenging. It is clear that where endometriosis-related infertility is concerned, the asymptomatic nature of the underlying disorder complicates things. This harbors the risk of a false unexplained infertility diagnosis and subsequent futile in vitro fertilization (IVF) attempts [1,2]. It is estimated that 30–50% of women with endometriosis are infertile [3], while it has been suggested that 20–50% of subfertile women are diagnosed with endometriosis [4,5]. Investigating the possibility of underlying endometriosis in patients presenting with undiagnosed infertility has made a case for benefitting the patient and ascertaining efficient management while avoiding IVF overuse [1]. Including the investigation of endometriosis in the infertility examination protocol has been suggested as a last step in the infertility investigation [1]—albeit this matter still raises a heated debate. 

The conundrum on how the reproductive potential of a patient with endometriosis is compromised has been thoroughly discussed in the literature. Several studies have suggested that fertilization rate, the number of competent embryos, blastocyst formation rate, implantation rate and pregnancy rate are impaired in cases of diagnosed endometriosis [6]. Clinical expressions and molecular manifestations of endometriosis range from hormonal imbalances and immunological responses to impairments in endometrial receptivity, to diminished gamete and embryo competence. The undoubtable and inevitable impact of endometriosis in cell function and developmental dynamics of the reproductive tissue has prompted researchers to investigate certain patterns showcasing how endometriosis detrimentally impairs fertility. Adhesions, chronic inflammation, disturbed folliculogenesis, luteal phase disruption, anti-endometrial antibodies, spermatozoa defects, progesterone resistance and disrupted uterotubal motility constitute some of the proposed mechanisms [7]. The question raised is which mechanisms are identified as the defining causative factors of infertility in endometriosis patients, and how, and to what extent these mechanisms lead to infertility [6]. In an effort to decipher and expose the true causative underlying factor or the one with the highest contributive significance in endometriosis-related infertility, a milestone study was performed by Simon et al, indicating that when endometriosis patients receive donor oocytes from fertile donors, the implantation and pregnancy rate was similar to oocyte donation patients without endometriosis. Along these lines, it was further documented that receiving oocytes originating from patients with endometriosis results in reduced implantation rate, suggesting the detrimental effect of endometriosis on oocyte and embryo quality more so than on endometrial receptivity [8]. Furthermore, a strong connection between the stage of endometriosis and the effect it exerts on embryo quality has been proposed, albeit there is substantial heterogeneity and discrepancies amongst the observed clinical findings [9]. Amongst the usual suspects, the deteriorated oocyte quality and subsequent impaired embryo quality have been proposed as two vital parameters that merit investigation in endometriosis related infertility [10].

Since oocyte and embryo quality are the two pillars of a successful IVF outcome, patients undergoing assisted reproduction with severely impaired oocyte and embryo quality due to endometriosis may be predestined to fail. In light of the lack of thorough mapping of the underlying molecular mechanisms responsible for the pathophysiology of endometriosis-related infertility, our understanding is restricted. This fact instigates a domino effect revealing weaknesses and limitations regarding the diagnosis, and management of infertile women presenting with endometriosis. Where endometriosis-related infertility is concerned more information on the molecular footprint of the impacted oocyte and embryo could serve as a more individualized predictor and means of evaluation. In the present review of literature, we focus on presenting the molecular pathways involved in endometriosis which may activate mechanisms that impair oocyte’s fertilization capacity and as well as subsequent embryo’s developmental capacity under the influence of a chronic inflammation. Providing insight from the molecular front of the oocyte and embryo perspective, is of critical value in decision making. Such data is required to orientate future research, emphasize on promising theories and prioritize them accordingly. 

## 2. Materials and Methods

A comprehensive review of the literature was performed in PubMed/Medline, Embase, and Cochrane Central databases up to December 2020. The keywords employed and combined for the search strategy were: ‘’Endometriosis’’, ‘’Endometrioma’’, ‘’Ovarian Endometrioma’’, ‘’Endometriosis Related Infertility’’, ‘’Endometriosis Pathophysiology’’, ‘’Endometriosis Pathology’’, ‘’Embryo Quality’’, ‘’Embryo Developmental Arrest’’, ‘’Oocyte Quality’’, ‘’Oocyte Maturation’’, ‘’Laparoscopy’’, “In Vitro Fertilization”, “Assisted Reproduction”, “Assisted Reproduction Techniques”, “Medical Assisted Reproduction”. The search was limited to full-length manuscripts published in English in peer-reviewed journals. Original research articles describing studies performed in both humans and animal models as well as review papers concerning the impact of endometriosis on oocyte quality and embryo developmental capacity were sourced. Following literature review, authors identified three major aspects via which endometriosis compromises oocyte quality and embryo developmental potential namely, increased reactive oxygen species (ROS) and free radical production; immune system dysregulation; impaired extracellular matrix remodeling and meiotic spindle distribution. A critical analysis on these three aspects was performed highlighting the major pathophysiological mechanisms via which endometriosis affects oocyte quality and embryo developmental dynamic.

## 3. Reactive Oxygen Species (ROS) 

A pivotal physiological process for embryos is the metabolism of molecular oxygen with a subsequent ROS production. ROS constitutes the side products of aerobic respiration and a natural part of cellular metabolism. Gametes, embryos, and their surroundings could constitute potential sources of ROS emissions. The categorization amongst endogenous and exogenous factors responsible for ROS generation allows for better understanding of the physiological processes involved in this event. Endogenous sources include numerous metabolic pathways and enzymatic activation on a cellular level, where the substantial contribution of several oxidases namely OXPHOS, xanthine oxidase and NADPH located in oocytes and embryos should be highlighted. Exogenous sources inducing ROS production include the oxygen concentration surrounding the developing embryo, metallic cations, visible light, and amine oxidase, as well as the ROS generated by spermatozoa which exert an equally detrimental effect on embryos [11]. These normal oxidative metabolic procedures are expected to produce ROS in cells; therefore, a safety mechanism has evolved to counteract this risky event of ROS emission—the production of antioxidants. An imbalance between free radicals and antioxidants results in detrimental oxidative stress [12]. Oxidative stress occurs when antioxidant mechanisms lack the ability to balance the production of ROS, which is the most crucial type of free radicals produced in mammalian systems. Oxidative stress may be a part of not only the pathogenesis of endometriosis but also of the progression of the disease, causing locally impaired tissue and cellular dysfunctions [13]. Nonetheless, one should not fail to highlight the beneficial role of ROS which-as previously mentioned-are an essential part of physiological processes. They participate in mediating signaling of normal development in healthy tissues, differentiation and apoptosis. Their regulatory functions in the reproductive tissue involve the regulation of folliculogenesis, oocyte maturation, the dissolution of corpora luteal, the implantation process and embryonic development [14,15]. While excessive expression of ROS is detrimental, inability to increase the levels of oxidative stress could also result in impaired cellular development and dysregulation [16].

### 3.1. How Elevated Levels of ROS Are Established in Endometriosis 

The correlation between endometriosis and ROS has been thoroughly investigated in literature [12]. The increased production of ROS by endometriotic lesions is attributed to the elevated proliferation rate that endometriotic cells exhibit [13]. In an effort to delineate the role of ROS in endometriosis-related infertility, it should be noted that the imbalance between ROS and antioxidants promotes an influx of oxidative stress agents in the peritoneum, follicular fluid, and ovaries [17]. As demonstrated, endometriotic cells present an altered phenotype of elevated ROS production which besides their association to infertility, they are considered responsible for the spreading of the endometriosis [13]. As a result, oxidative stress biomarkers are significantly increased in patients presenting with endometriosis [12]. ROS also constitute proinflammatory mediators and a key component of chronic inflammation conditions. Considering the aforementioned, we conclude that the altered ROS production originating from endometriotic lesions, could detrimentally affect the microenvironment of the fallopian tubes, the growing follicles as well as the oocyte and embryo quality via direct dysregulation of the cellular processes or indirect via inflammatory phenomena [6]. 

### 3.2. Mechanisms Indicating the Role of ROS in Cellular Damage

Another interesting aspect worth reporting on regarding the theory of the retrograde menstruation into the peritoneal cavity is that the presence of numerous lysed erythrocytes of the refluxed menstrual blood results in elevated iron levels which are a source of free radicals production in vivo [18,19]. Due to their highly unstable and reactive state, when in abundance, they could damage cellular organelles and crucial molecular components of the cell [6]. What is more, intense hemoglobin destruction and release of iron load leads to the production of hydroxyl radicals which are the most reactive free radical species affecting amino acids, purines and pyrimidines, as well as membrane lipids, initiating a domino of effects known as lipid peroxidation [20]. Malondialdehyde (MDA) is employed as an indicator for lipid peroxidation and therefore a marker of oxidative stress. It has been indicated that its levels are increased in the follicular fluid originating from patients presenting with endometriosis in comparison to individuals without endometriosis [19]. In addition, data suggest that in patients presenting with endometriosis, the cellular antioxidant mechanisms are significantly impaired. Studies indicate that both glutathione-*S*-transferase’s (GST) concentration and functionality are compromised, highlighting that their total antioxidant capacity is mitigated [21,22].

The excessive production of ROS, also affects certain molecular components in the cells, namely adenosine triphosphate (ATP) synthesis or intracellular calcium ions’ concentration. This in turn prompts a series of events interfering with numerous cellular functions [23]. Initially, the depletion of ATP due to mitochondrial DNA damage results in reduced production of guanosine triphosphate (GTP) and reduced phosphorylation of microtubule-associated proteins (MAPs) which are vital for assembling microtubules and maintaining the required dynamic instability [24,25]. Furthermore, the ratio of reduced glutathione (GSH) and oxidized glutathione (GSSG) -which is employed as a biomarker of oxidative stress- is affected, contributing in the polymerization-depolymerization process, while the elevated calcium levels inhibit polymerization and enhance depolymerization of microtubules [23,24]. Also, ROS could directly inactivate enzymes involved in the activation of MAPs which maintain the dynamic equilibrium of microtubules and target DNA inducing chromosomal aberrations as it has been documented on a hamster model [26]. 

### 3.3. The Impact of ROS in Oocyte and Embryo Competence

As demonstrated, ROS exert a detrimental impact in several aspects of reproductive physiology namely oocyte maturation, ovarian steroidogenesis, ovulation process, implantation capacity, and blastocyst formation. The cytotoxic effects of ROS include peroxidation of membrane phospholipids, leading to increased permeability of the cell membrane, impaired cell membrane integrity, enzyme activation and dysfunction along with severe deterioration in DNA structure, and subsequently cell death [27,28]. Regarding ROS’ effects on oocytes, it has been demonstrated that in mice the disassembly of meiotic spindle microtubes is attributable to the disruption of the dynamic equilibrium of microtubules by dysregulation of tubulins dimers -the subunit of microtubules. As the free radical theory of ageing suggests, this impact on the dynamic instability of microtubules and their motion forces, results in incorrect segregation of chromosomes during meiotic divisions [23,29]. Such an unstable state of incorrect chromosomal segregation of the genetic component leads to aneuploidy, impairing reproductive dynamics. Oxygen free radicals is believed to be negatively correlated to embryonic development and dynamics [28]. Interestingly, it has been documented in studies performed employing mouse embryos, that embryo arrest caused by ROS could be reversed when culture media were supplemented with a key component of the antioxidant system called superoxide dismutase [30]. The extent of embryo damage caused by the generation of ROS is unpredictable with numerous compromised cell functions due to the fact that ROS diffuse through cell membranes and interact with several cell molecules [11]. Embryotoxicity and teratogenesis have been further described [31], enriching the long list of factors hampering developmental competence of embryos due to this state of increased oxidative stress. Damage by ROS is also caused due to lipid peroxidation which is an oxidative destruction of polyunsaturated fatty acids that compose plasma membranes [32]. This phenomenon increases membrane permeability, degradation in membranes’ integrity, enzymes’ disruption and inactivation, as well as severe damages in the structure of DNA. In such cases, cells death is inevitable [33]. Due to lipid peroxidation induced by ROS, cell division, metabolite transfer and mitochondrial function are compromised as well. Furthermore, the effect ROS exerts on proteins is detrimental, promoting cell cycle dysregulation, while DNA fragmentation in the nucleus has been described provoking events of embryo developmental arrest [34]. Mitochondrial DNA is further affected by the presence of ROS in the cell, as it is susceptible to mutations. Metabolic dysfunction is described which also contributes to embryo’s developmental arrest and occasionally, in apoptotic events due to metabolic alterations. 

**Figure 1:**
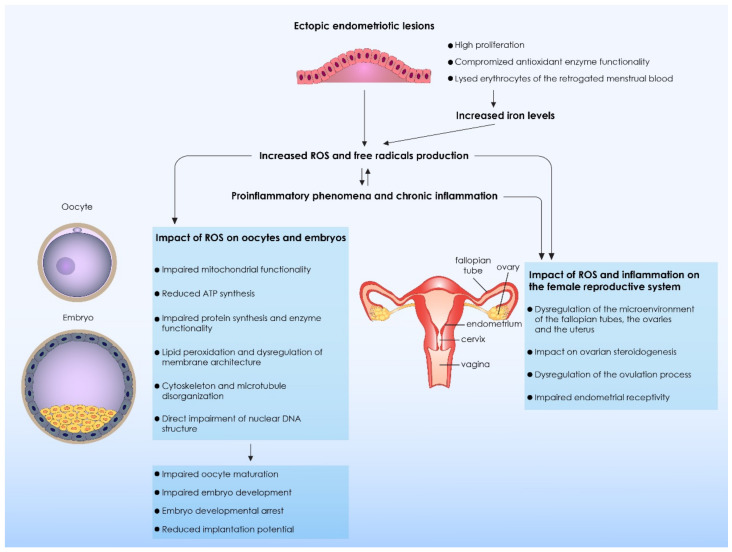
The impact of reactive oxygen species (ROS) and free radicals on oocyte quality and embryo developmental capacity. Ectopic endometriotic cells are characterized by a high proliferation and are presenting with compromised antioxidant enzyme functionality. As a result, ROS production and secretion from ectopic endometriotic lesions are significantly increased. Moreover, erythrocytes originating from the retrograde menstrual blood are lysed, thus an increased amount of iron molecules is released. Iron levels constitute a source of free radical production in vivo, leading to a further increase of ROS and free radicals. The Increased ROS and free radical production leads to the stimulation of proinflammatory phenomena and as a result chronic inflammation is established. The ROS have a direct detrimental impact on oocytes and embryos. Several molecular and cellular processes are compromised, resulting to the impairment of oocyte maturation and embryo development. Thus, embryo developmental arrest and reduced implantation potential are commonly observed in patients presenting with endometriosis. Moreover, extensive ROS production and immune system dysregulation both detrimentally affect the functionality of the female reproductive system. The ovulation process as well as ovarian steroidogenesis are significantly dysregulated. A significant impairment of the microenvironment of the fallopian tubes, the ovaries and the uterus is also observed, leading to impairment of endometrial receptivity. ROS: Reactive Oxygen Species; ATP: Adenosine Triphosphate; DNA: Deoxyribonucleic Acid; MAP: Microtubule Associated Proteins.

Endometriosis is accompanied by elevated levels of ROS. Therefore, several considerations are raised as to the extent that these byproducts of metabolism complicate physiological processes leading to subfertility or even to infertility. Interestingly, it has been suggested that ROS effect on bovine’s embryo’s implantation dynamic depends on the developmental stage during which the embryos exposed to ROS. Zygotes and blastocysts are considered to be particularly sensitive and not resistant to the toxic effects of exogenous H_2_0_2_ [35]. In vivo defense mechanisms protecting embryo development from ROS, have been described, including antioxidant agents, repair mechanisms and molecular pathways disrupting the formation of ROS at the initial stages of formation [36]. Based on these observations, the question in regard to what the optimal duration of embryo culture should be for endometriosis patients is raised. On one hand, in the controlled conditions that in vitro culture provides, embryos are not exposed to the influx of ROS production as is the case in the peritoneal fluid in vivo, despite the ROS production of the embryo’s own metabolic activity [14] which is limited. On the other hand, these safe culture conditions and deprivation of ROS exposure, does not allow the embryo to develop any defense strategies and present repair mechanisms for its protection against ROS. Should these endometriosis-affected embryos be transferred earlier in the uterus to achieve adaptation? Could that contribute towards development of repair potential and further establish valuable interactions with the endometrium which is a prerequisite for a successful implantation? Or should retaining them in an environment of reduced ROS and mitigated epigenetic alterations be considered the optimal line of approach? Measurement of oxidative stress markers such as iron and nitric oxide could be employed as non-invasive diagnostic tools [37], therefore their identification should be prioritized by researchers [38]. Furthermore, the beneficial role of anti-oxidant agents in the prevention and treatment of endometriosis should be further explored [37], while clinical trials are required to determine their efficiency [17]. Overall, thorough investigations of how oxidative stress intertwines with endometriosis-related infertility remain to be conducted and communicated back to the practitioner. However, the accumulated modifications in numerous cell functions that are undoubtedly linked to intense apoptotic events help to form an adequate explanation serving as the basis for future research and conclusions. An overview of the mechanisms involved in the production of ROS, as well as the detrimental effect they exert on oocyte and embryo quality is presented in Table 1 and Figure 1, respectively. 

## 4. Dysregulation of the Immune System 

### 4.1. The Disruption of the Immune Balance in Endometriosis 

The complexity of the immune system allows for its mechanisms to maintain homeostasis and restore balance in physiological processes when disrupted. In order for endometriosis to manifest, it has been documented that endometrial cells emerge in ectopic areas, in places where the immune system lacks the ability to detect and eliminate them, mistakenly allowing them to implant and grow [39]. In a nutshell, manifestation of endometriosis could be attributed to the endometrial cells’ capacity to modify immune tolerance. Since these cells retain their hormonal responsive ability and are periodically active, they may initiate an inflammatory response which eventually is established as a chronic state [7]. An influx of helper and regulatory T-cells are observed in the initial acute phase of inflammation. For the chronic phase to be established, macrophages are recruited which in turn invoke the formation of adhesions and angiogenesis. As evidenced by published data, cytokines, chemokines and prostaglandins, constituting pivotal mediators of inflammation, are detected in increased levels in the peritoneal fluid in cases of endometriosis [40]. Besides the key role of ROS, cytokines of the peritoneal fluid have been proposed to further interfere with reproduction [41] since endometriosis facilitates a buildup of inflammatory regulators and signaling agents. What is more, despite ROS contribution as secondary messengers of signaling pathways, they may also act as regulators of gene expression producing cytokines and numerous regulators of the immune response [42]. Vice versa, the state of inflammation established in endometriosis evokes a persistent immunological activation resulting to an enhanced presence and copious production of ROS, along with the numerous antigens expressed by cells in the endometrium. 

Peritoneal fluid constituents are cells participating in the immune response, controlling the environment gametes and embryos are exposed to [41]. Normally, peritoneal fluid contains predominately macrophages, lymphocytes, eosinophils and mast cells within a normal concentration range. Endometrial cells promote inflammation and therefore activation of macrophages [43]. In patients with endometriosis, macrophages in the peritoneal fluid are activated, and enter a state of secreting cytokines. As to why these macrophages exhibit such an intense activation in the peritoneal fluid of patients with endometriosis, it was proposed by Halme, that the enhanced production of macrophage-derived growth factor in the peritoneum of endometriosis patients, results in maturational changes for the macrophages [44]. The macrophage activation is more pronounced in patients with mild endometriosis [45], which dictates further research on the immunological aspect of the disease. A primary regulator of immune response is Interleukin-1 (IL-1) which induces secretion of prostaglandin. Macrophages hold the potential to secrete IL-1 during the fertilization process and early embryo cleavage stage process which may exert a detrimental effect in reproductive capacity [45]. Interestingly, the activity of IL-1 is higher in patients with more severe cases of endometriosis, whereas in normal fertile women IL-1 is not detectable. What is more, IL-1 is associated with fibroblast proliferation, collagen deposition and formation of fibrinogen, allowing for a hypothesis to be drawn as to how fibrosis and adhesions occur in advanced stages of endometriosis [45]. To summarize the role of IL-1 and highlight the pathways through which it may disturb the reproductive capacity, several points should be highlighted. It stimulates production of IL-2 from T-cells which in turn represents the stimuli for clonal expansion of various types of T-cells and it enhances natural killer cell activity [46]. Also, it stimulates B-cell proliferation and the production of antibodies [47]. Elevated levels of TNF-a, IL-8, and IL-10 are also detected in women with endometriosis. The source of these molecules is considered to be the macrophages of the peritoneal fluid along with the cocktail of cytokines they produce [48]. VEGF levels are associated with follicular health and vascularization [49], therefore its reduced levels in endometriosis may be linked to reduced embryo quality [50]. All these proposed damage mechanisms add another level of complexity in understanding the role of immune system in fertility.

### 4.2. The Impairment of the Reproductive Status Due to Affected Immunological Parameters

Investigation of the immune system sheds light on another perspective of endometriosis-related infertility. The severe established case of chronic inflammation in patients with endometriosis may affect fertility potential in numerous ways. The enhanced levels of several cytokines in the follicular fluid including interleukin-1β, IL-8, IL-10 and TNF-α may be linked to impaired ovarian response [51]. Prostaglandins and cytokines could deteriorate the embryo’s developmental capacity and implantation dynamic [52], while the dysfunction in the HPO axis and the subsequent disturbance in the orchestration that regulates follicular developmental capacity and oocyte maturation could detrimentally impair fertility [53]. Damage in the initial stages of gametogenesis until the migration of oocytes to the uterus is attributed to the cytokines produced by the activated macrophages. Macrophages activation can deteriorate metaphase II mouse oocytes due to the production of oxidizing agents by myeloperoxidase—a pre-inflammatory enzyme, while cumulus cells lack the ability to provide protection [54]. A plausible theory to interpret the impaired oocyte and embryo quality in endometriosis patients is the presence of inflammation in the female reproductive organs. Intra-follicular levels of IL-8, IL-12 and adrenomedullin are increased in women with endometriosis and constitute key intrafollicular prognostic indicators of diminished reproductive dynamic of both the oocytes and the embryos [55]. A negative association between IL-12 and folliculogenesis, oocyte quality, and implantation has been voiced, as well as TNF-a elevated levels seem to be correlated with poor oocyte and embryo quality [56,57,58,59]. Modifications of the granulosa cell cycle in endometriosis may be attributed to the cytokine’s observed changes [60]. Elevated levels of IL-10 have been described to causing arrest in the G0 phase due to the downregulation of p27 [61], coupled with the cocktail of cytokines namely IL-6, IL-1β, IL-8 and IL-1α, crucial cell cycle abnormalities and dysfunction may be noted [62]. On another note, endometriosis could further cause dysregulation of heat shock proteins which are protecting cells from oxidative stress and inflammation. Their role as molecular chaperones is to assist the folding of newly synthesized proteins. Interestingly, a direct connection with the immune system has been proposed due to antigen processing, presentation and peptide binding. Therefore, in endometriosis where immune responses are affected, the abnormally increased expression of heat shock proteins triggers events of apoptosis caused by the cellular injury they inflict [63]. 

In an advanced stage, intensified apoptotic events are detected, compromising oocyte quality in patients presenting with endometriosis. Molecular mechanisms involving the immune response that may interfere with the ovulatory process promote dysregulated steroid ovarian secretion or poor luteal function [39]. This dysfunction is attributed to the diminished aromatase activity in granulosa-lutein cells as well as to the compromised progesterone production and impairment of steroidogenesis overall. Under these circumstances, corpus luteum formation and functionality is jeopardized and this inevitably leads to subfertility [64]. When murine embryos were incubated in peritoneal fluid form endometriosis patients, growth rate was decreased, while DNA fragmentation and an enhanced rate of apoptosis, and arrested embryos were documented by several studies [65,66]. This cumulative collective evidence could serve as arguments buttressing the idea that peritoneal fluid in women with endometriosis detrimentally effects embryo dynamic due the inflammation induced embryotoxicity -especially by TNF-a [67]. To further underline the detrimental effect of endometriosis, immune cells recruited in the peritoneal fluid of patients with endometriosis experience a process of “oxygen explosion” during which superoxide anion and hydrogen peroxide are released massively, damaging the tissue and forming adhesions [68]. 

**Figure 2:**
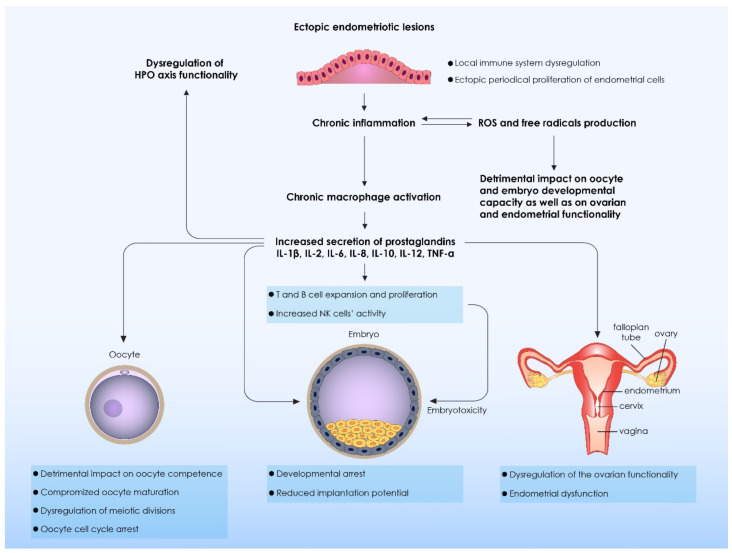
The impact of disruption of the immune system balance on oocyte quality and embryo developmental capacity. Immune system dysregulation is observed in the areas where ectopic endometriotic lesions are localized. As a results a periodical proliferation and activation of the ectopic endometrial cells is observed. High proliferation levels lead to an increased production and secretion of ROS from the ectopic endometriotic lesions. The increased ROS and free radicals production leads to the stimulation of proinflammatory phenomena and as a result a chronic inflammation is established. Endometrial cells promote inflammation and therefore a chronic activation of macrophages in the peritoneal fluid. Activated macrophages secrete a high amount of cytokines and prostaglandins. From one hand, secreted cytokines induce lymphocytes’ and natural killer cells’ (NK) activation and proliferation. These activated immune cells directly affect embryo development inducing embryotoxic phenomena and leading to embryo developmental arrest. Affected embryos are characterized by a significantly reduced implantation potential. On the other hand, cytokines and prostaglandins directly affect oocyte and embryos, jeopardizing oocyte competence and oocyte maturation. As a result, inflammation leads to dysregulation of meiotic divisions, inducing oocyte cell cycle arrest. Additionally, cytokines directly affect HPO axis regulation as well as ovarian and endometrial functionality affecting the ovulation process and endometrial receptivity, respectively. ROS: Reactive Oxygen Species; IL: Interleukin; TNF-a: Tumor Necrosis Factor a; HPO axis: Hypothalamus Pituitary Ovarian axis.

It becomes evident that this vicious cycle of inflammation releasing ROS and ROS contributing to the release of cytokines that in turn orchestrate inflammation should be further explored, and interpreted as the complex nature of these mechanisms and the interrelationships that bind them may hold the key in providing vital information on endometriosis related infertility and how to manage it. Several studies investigating the isolation of peritoneal fluid from patients with endometriosis and its implementation in culturing mouse embryos, have contributed contradicting data. On one hand it is indicated that the developmental capacity of these embryos may be impaired, while others report no adverse effects. This contradiction leaves room for variant interpretations and calls for further investigation. However, it appears that there is value in following on the hypothesis that the preimplantation embryo of patients with endometriosis is exposed to a toxic environment attributed to the intraperitoneal fluid. Considering the three pillars, namely that apoptosis in granulosa cells is a tool to assess the quality of oocytes, coupled by the fact that endometriosis patients present with the highest prevalence of apoptotic granulosa cells compared to fertile patients [60], and finally taking into account that a causative factor of apoptosis is cytokines, it becomes evident that the effect of cytokines on oocyte and embryo quality merits further investigation and may well provide significant answers. An overview of the mechanisms involved in the dysregulation of the immune system, as well as the damage it exerts on oocyte and embryo quality is presented in Table 2 and Figure 2, respectively.

## 5. Meiotic Spindle Disruption and Extracellular Matrix Remodeling 

### 5.1. How Endometriosis Affects the Cellular Kinetics and Chromosomal Integrity

Amongst the multiple pathways explored to define the culprits of endometriosis-related infertility, alterations in meiotic spindle that promote increased rates of aneuploidy have been proposed. Nuclear and cytoplasmic impairment, namely cytoplasmic fragmentation and uneven cleavage have been described as findings indicative of endometriosis. What is more, nuclear and cytoplasmic maturation are essential for a successful fertilization. Any disturbances regarding these procedures could lead to morphologic and genetic abnormalities. Poor quality oocytes incurring such anomalies are accompanied by reduced fertilization and implantation rates [69]. In turn, the significant DNA damage that oocytes experience results in higher frequency of aneuploidy, while the duration of exposure to the impaired peritoneal fluid is correlated with the extent of the damage caused [70]. 

### 5.2. Meiotic Spindle Disruption and Consequences on Oocyte and Embryo Competence

The spindle apparatus is a fundamental component for securing correct segregation of the genetic material performed during meiotic divisions, while it maintains chromosomal organization. Spindle’s integrity is also considered as a potential marker for oocyte quality and dynamic [71]. Spindle cell complex of oocytes is prone to microtubule dysregulation and meiotic errors, buttressing the theory of increased aneuploidy rates in endometriosis [72]. When mice oocytes are exposed to peritoneal fluid of endometriosis patients, severe disruption in microtubules and chromosomes is detected, potentially attributed to alterations in the meiotic spindle [71], attributed to causative factors that comprise the peritoneal fluid. Studies focusing in defining these factors, indicated that the meiotic spindle is affected by increased levels of cytokines as well as by increased levels of oxidative stress; both constituting predominant characteristics of endometriosis as described above [73]. Experiments on animal models suggest that among the factors that target the meiotic spindle is the exogenous exposure to hydrogen peroxide and TNF-a [74]. Following exposure to recombinant mouse IL-6, disruption in microtubule and chromosomal alignment of MII mouse oocytes has been documented [75]. 

Chromosomal organization, along with the formation of the second polar body depend on the meiotic spindle [76]. A meiotic spindle disruption promotes events of chromosomal dispersal and arrest of the embryo development [77]. The occurrence of meiotic anomalies in the oocyte results in inability to achieve normal fertilization, or to achieve developmental competence as an embryo in case fertilization is achieved [78]. Furthermore, the first polar body may exhibit signs of abnormalities in an increased frequency. These abnormalities include delay in extrusion and impaired polar body divisions in patients with endometriosis [79]. In animal models, the potential abnormalities observed may also include spontaneous oocyte activation, the formation of pseudopronuclei, and karyomeres enclosed by a de novo formed nuclear envelope [80]. The incidence of aneuploidy is somewhat a field of contradictions, since opposing evidence are documenting either equal or increased rates of aneuploidy in patients with and without endometriosis [72,81].

### 5.3. Extracellular Matrix Remodeling Affected by Endometriosis

Matrix metalloproteinases (ΜMPs) are a family of proteins associated with structural cellular events, namely the degradation of extracellular matrix (ECM) and components of basement membrane [82]. The extracellular matrix participates in crucial physiological events such as cell proliferation and differentiation. Therefore, any factor capable of modifying the ECM could exert a detrimental impact to normal as well as pathological events. Especially, in cases of extensive ECM remodeling such as embryogenesis, the role of MMPs as matrix-degrading agent is highlighted. For normal follicular and embryo development, extracellular matrix remodeling by MMPs constitutes a prerequisite [82]. A potent regulator of MMPs is cytokines, therefore as expected, in the peritoneal fluid of patients with endometriosis, elevated levels of MMPs and decreased levels of its inhibitors are detected [83]. MMPs’ controlled proteolysis is regulated by tissue inhibitors of metalloproteinases (TIMPs), and both are required for orchestrating the remodeling of the ECM [84]. Besides follicular and embryo development, ovulation and formation of the corpus luteum are processes that require extensive remodeling. As suggested, the alterations in the ovarian ECM during the delicate process of folliculogenesis are performed mainly by the MMP system [85]. Studies performed in animal models mimicking endometriosis pathogenesis indicate that TIMP1 which is secreted by endometriotic lesions directly into the peritoneal fluid holds the potential to localize in the theca of preovulatory follicles. This results in alteration in the MMP activity disrupting the process of ovulation and the overall ovarian function [80]. Following ovulation, the oocyte complex is exposed to the peritoneal fluid flooded with abnormal TIMP1 concentrations. This abundance of TIMP1 exerts a detrimental effect in the oocyte, since it can infiltrate into the nucleus and disrupt the overall cell cycle hindering the oocyte’s development until the stage of the preimplantation embryo [80,86]. 

**Figure 3:**
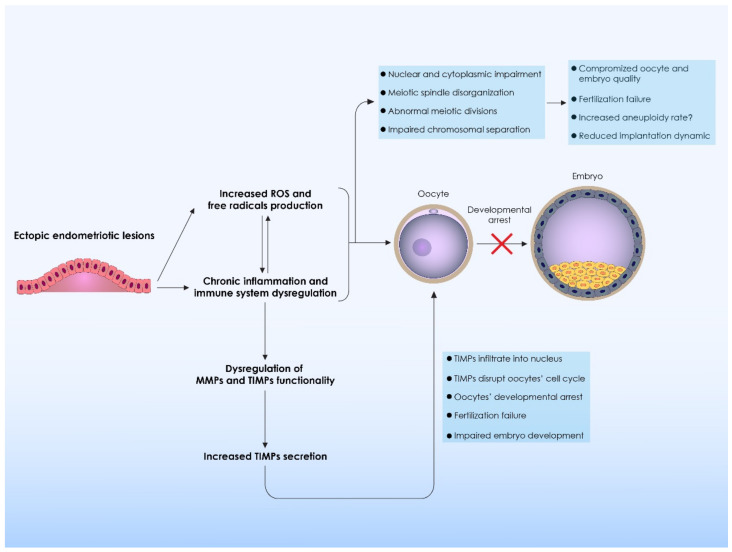
Mechanisms involved in oocyte meiotic spindle disruption and extracellular matrix remodeling. Immune system dysregulation is observed in the areas where ectopic endometriotic lesions are localized. As a result a periodical proliferation and activation of the ectopic endometrial cells is observed. High proliferation levels lead to an increased production and secretion of ROS and free radicals from the ectopic endometriotic lesions. The increased ROS and free radicals production leads to the stimulation of proinflammatory phenomena and as a result a chronic inflammation is established. Both ROS and chronic inflammation directly affect the molecular and cellular functions of the oocytes. Nuclear and cytoplasmic impairment is observed, leading to meiotic spindle disorganization, which in turns leads to abnormal meiotic divisions, impaired chromosomal separation and oocyte developmental arrest. These phenomena compromise oocyte and embryo quality, leading to fertilization failure and reduce embryo implantation dynamic. It has been voiced that meiotic spindle disruption observed in oocytes originating form patients with endometriosis could increase aneuploidy rates, however this does not constitute an established knowledge. Chronic inflammation also affects extracellular matrix remodeling, affecting MMPs and TIMPs production and functionality. As a results an increased secretion of TIMPs is observed. TIMPs directly infiltrate the oocyte’s nucleus, disrupting oocyte’s cell cycle. Thus, TIMPs disorganize oocyte physiology, leading to developmental arrest, fertilization failure and impaired embryo development. ROS: Reactive Oxygen Species; MMPs: Matrix metalloproteinases; TIMPs: Tissue Inhibitors of Metalloproteinases.

The puzzling issue of connecting the dots between endometriosis and infertility extents into the perplexing contribution of cellular kinetics. Microtubule and chromosomal changes have been observed in patients with endometriosis, adding another level of complexity to the mechanisms entailed in the infertility manifested. Available evidence is sourced from bovine and mouse oocytes suggesting that chromosomal and spindle misalignments could occur in cases of endometriosis, however as aptly commented by Mate, the extrapolation to the human physiology requires more concrete data. Less recent studies demonstrating increased aneuploidy rates present with certain limitations that pose a great challenge when aiming to draw safe conclusions. On the other hand, recent studies proposing that there is no association between aneuploidy and endometriosis present with more robust data [6]. It should be noted that studies investigating the incidence of aneuploidy in endometriosis and the respective mechanisms entailed, are limited by default. Investigating the incidence of aneuploidy itself presents with an inevitable limitation: namely that the inflammatory milieu is accompanied by an influx of cytokines that cannot be properly defined nor excluded from the equation. An overview of the mechanisms involved in the meiotic spindle disruption, as well as on extracellular matrix remodeling leading to impaired oocyte and embryo quality is presented in Table 3 and Figure 3, respectively. 

## 6. Discussion

As the Practice Committee of ASRM aptly commented in 2012, “*the causal relationship between endometriosis and infertility has not been clearly established*” [87]. The inability to identify a precise molecular mechanism that elucidates the events prior, during and after manifestation of endometriosis, portrays the complexity in diagnosing and managing the disease. It becomes evident that the extreme of pathophysiological events occurring in an endometriosis-induced environment impair the overall state of molecular balance fueling a vicious circle. The events engaging in manifesting reduced fecundity when endometriosis is established are chaotic yet connected. In this critical analysis of literature, authors describe the plethora of events that unfold and present how they potentially impair oocyte and embryo dynamics.

Overall, literature findings suggest that one of the primary causative factors for infertility in endometriosis is the limited number of matured oocytes [9], accompanied by hormonal imbalances referring to estrogen synthesis and progesterone resistance [88]. However, following review of the literature it is undoubtedly showcased that the physiological events that oocytes and embryos are exposed to should be considered in the manifestation of endometriosis-related infertility. Employing time-lapse technology enabled researchers to detect altered morphokinetics and poorer embryo quality in patients with endometriosis [89]. Many studies have focused on exploring the synthesis and effect of peritoneal fluid in order to decipher its influence on the progression of the disease and the potential pathophysiological mechanisms resulting in endometriosis-related infertility. Reactive oxygen species exert a detrimental effect in cellular functions as they hold the potential to impair protein activity and dysregulate gene expression [90]. The role of oxidative stress in endometriosis has been thoroughly investigated as a potential factor contributing to the onset of endometriosis. Oxidative stress has been identified as a component of the inflammatory response [36], that is further responsible for the disturbance in meiotic spindle architecture [74] and oocyte degeneration [32]. Any alteration in functionality of the cytoskeleton and disorganization of the meiotic spindle could serve as an adequate explanation for a reduced fertilization rate and impaired embryo development as it has been indicated in a study investigating the impact of cryopreservation on mouse oocytes [77]. The inflammatory milieu along with the cytokines released contribute to deterioration of physiology to the point of establishing endometriosis and infertility. The so called “burn-out effect” on the ovarian reserve due to the toxic surroundings caused by the endometriosis-induced inflammatory state [91] could serve as a solid causative factor for endometriosis-related infertility. This phenomenon induces fibrosis, defects in vascularization and loss of cortex-specific stroma that ultimately result in compromised follicular maturation and intensified atresia observed in follicles [91]. 

To further complicate the underlying mechanisms participating in the onset of endometriosis-related infertility, epigenetic changes may play a significant role. In surgically induced endometriosis in rat animal models with subsequent compromised oocyte and embryo quality and development, an intriguing conclusion was drawn that further expands our current knowledge on endometriosis. It was indicated that oocytes and embryos originating from the first generation of the rats which did not undergo any surgical intervention exhibited anomalies in oocytes and preimplantation embryo development similarly to their mother with induced endometriosis. What is more, it was reported that the first generation presented with reduced fecundity. This constitutes the first report in literature showcasing that maternal exposure to endometriosis exerts a negative impact on the oocyte and embryo quality and impacts developmental capacity in subsequent generations. Therefore, the worrisome argument on the permanent epigenetic changes caused by endometriosis in subsequent generations appears to be valid and merits further investigation in humans [80]. 

Several meta-analyses support the notion that reduction in fertilization and implantation rates are expected in women with endometriosis [9], and a meta-analysis performed by Barbosa indicated that the number of yielded oocytes was reduced in endometriosis patients [92]. Other studies indicate that endometriosis severity is not directly associated with the compromised fertility status. It has been reported that patients with endometriosis present with a lower number of mature oocytes, as well as reduced fertilization rates in cases of minimal/mild endometriosis compared to moderate/severe [93]. What becomes evident is that patients presenting with endometriosis constitute a population characterized by a high heterogeneity [9], which can serve as an explanation on the paradox clinical observations documented. Overall, only a few studies have approached the issue of endometriosis from the clinical embryology perspective, therefore essential data is missing [9]. The limited number of studies assessing clinical embryology data provide conflicting results that do not allow for any extrapolation. Recruiting a heterogeneous group of patients and studying various stages of the disease, while lacking control groups or strict inclusion criteria seems to result to these discordant conclusions. What is more, there is a discrepancy on the treatment protocols applied between studies implementing either a pharmaceutical or a surgical approach. To add to that, the assessment of IVF outcome is based on various parameters, lacking consistency [62]. The fact that, a successful outcome can be interpreted differently either as a successful implantation or a live birth, allows for no comparisons to be performed or robust conclusions to be drawn. Can we rely on the conclusions provided by these studies to shape and define the identity of endometriosis when there are so deafening limitations and discrepancies in design and execution? The many faces of endometriosis should be acknowledged, instead we misguidedly strive to fit all characteristics into a single strictly defined pathology. Perhaps, it is time for the heterogenous clinical findings to be interpreted based on the unique underlying molecular background. 

To elaborate on the limitations observed in current literature, data and evidence on the mechanisms impairing oocyte and embryo quality principally originate from animal studies. This constitutes a considerable limitation in investigating endometriosis. Researchers highlight the bioethical dilemmas raised by manipulating human genetic material and note the ethical restrictions in manipulating human embryos [67]. Thus, for research purposes, employing animal models is imperative for developing novel non-invasive diagnostic tools [94]. Extrapolating on the implications of endometriosis from animal models to humans should be exercised with caution [79]. Employing an animal model comes with certain limitations that stem from the fact that animals bear no genetic, immune or environmental susceptibility to exhibit signs of endometriosis [79]. What is more, the phenomenon of superovulation in animal models has been proposed to impair oocyte and embryo quality, and therefore it stands as a severe cofounder. Consequently, definitive statements on the true effect of endometriosis on human oocytes and embryos have yet to be concluded. 

In light of the lack of concrete data optimal management of endometriosis still raises heated debate. Investigating strictly one parameter in a multifaceted pathology seems to serve as a fundamental and inevitable limitation in published studies. The role of the endometrium along with the molecular pathways involved in patients with endometriosis is a promising field of study. Weighing the role of either the endometrium, or the quality of oocytes and embryos individually when they all are equally deteriorated in these patients may prove to lead to futile conclusions. In theory, what is required-yet not feasible-is an all-inclusive approach taking into account all aspects in this complicated issue. Nonetheless, proper research methodology allows for examining one parameter at a time and this in itself is the major conundrum here, as it is impossible to study in an isolated methodologically sound manner one parameter. 

Following the chain of intertwined events from diagnosis to treatment, it is the lack of robust and minimally invasive tools in detecting endometriosis, which fails to enable a timely and precise diagnosis which could in turn set the tone for an optimal treatment strategy. Following diagnosis, clinicians face a dilemma when managing a case of endometriosis-related infertility, since the optimal approach is yet to be defined. The available strategies include either subjecting the patient to an IVF protocol or proceeding with attempting to surgically treat endometriosis. Following surgical intervention to restore pelvic anatomical structures and remove endometriotic lesions, patients are invited to either initiate assisted reproduction treatment or conceive naturally when there is no other underlying pathological etiology detected. As aptly indicated by Taylor, the micro metastasis and all systemic effects may not be eradicated by simply removing signs of the disease [95]. Surgical treatment removes visible endometriotic lesions and restores anatomical structures, but is that always enough to restore fecundity? What if, surgically removing endometriotic lesions harbors the risk of impairing ovarian function? [96]. On the other hand, directly enrolling patients in an IVF protocol harbors the risk of IVF overuse, with all the psychological and financial repercussions entailed. Apparently, attempting to draw a definite conclusion on whether to treat ovarian endometriosis prior to IVF has to be further elucidated in the future [97]. 

Interestingly, there are no published studies reporting on the efficiency of different management strategies on improving oocyte and embryo quality in patients presenting with endometriosis-related infertility. Only indirect conclusions can be drawn with regard to the impact of different treatment strategies on oocyte and embryo quality by studying the IVF outcomes in patients presenting with endometriosis-related infertility. Discrepancies are observed between the practices all over the word with regard to the most efficient management protocols that patients should be subjected to in order to improve IVF outcomes in the context of better managing endometriosis related infertility. According to the European Society of Human Reproduction and Embryology (ESHRE) guidelines, which depict evidence-based clinical practice in Europe, surgical removal of endometriotic lesions via laparoscopy could enhance IVF outcomes in patients presenting with minimal endometriosis [98]. With regard to severe deep infiltrating endometriosis, ESHRE experts do not recommend surgical removal of endometriotic lesions prior to IVF because there are no robust evidence indicating a beneficial effect of this approach [98]. Considering ovarian endometrioma, ESHRE strongly recommends that clinicians should not procced to cystectomy prior to IVF treatment when ovarian endometriomas are greater than 3 cm, acknowledging the possible harmful effects of these approach on ovarian reserve [98]. Considering the respective guidelines provided from the ASRM, which influence evidence based clinical practice in the United States of America (USA), these guidelines provide unclear recommendations with regard to the usefulness of laparoscopic surgery prior to IVF treatment [87]. Interestingly and in contrast to the ESHRE guidelines, ASRM recommends surgical treatment of large endometriomas (greater than 4 cm) prior to IVF treatment in order to improve access to follicles during the oocyte retrieval procedure as well as to increase ovarian stimulation efficiency [87]. Despite the differences, both of these guidelines recommend that medical treatment including hormonal contraceptives, progestogens and GnRH analogues, do not constitute effective approaches towards improving fertility rates in patients presenting with endometriosis and thus should not be offered on this indication alone [87,98]. Guidelines provided from other organizations, including the Korean Society of Endometriosis are generally in the same line with the directions provided by ESHRE [99]. The guidelines provided by the Clinical Practice Gynaecology Committee and the Executive and Council of the Society of Obstetricians and Gynaecologists of Canada albeit they provide robust data, nonetheless, the do not provide specific guidance regarding to the optimal treatment protocol for managing endometriosis-related infertility [100]. Experts in Canada recommend that asymptomatic women with endometriosis should not receive any medical or surgical treatment to improve their reproductive potential [100]. In the same line with ESHRE’s guidelines, the Canadian gynaecology committee highlights that laparoscopy treatment on minimal or mild endometriosis could enhance pregnancy outcomes, in contrast to the severe endometriosis were surgical treatment’s benefits are unclear [100]. In contrast to the ESHRE and in the same line with the ASRM, laparoscopic excision of ovarian endometriomas greater than 3 cm is recommended in Canada [100]. Moreover, the role of the IVF treatment in patients presenting with endometriosis is unclear in Canadian guidelines [100]. The guidelines provided from the National Institute for Health and Care Excellence (NICE), shaping clinical practice for managing endometriosis-related infertility in the United Kingdom, recommend that women experiencing endometriosis should be advised to conceive naturally via regular unprotected sexual intercourse for at least two years prior to embarking on IVF treatment [101]. The NICE guidelines also recommend that patients presenting with endometriosis should not receive medical treatment, because this practice does not improve spontaneous pregnancy rate [101]. In the same guidelines authors note that laparoscopic excision or ablation of endometriosis lesions as well as adhesiolysis is recommended, as this practice enchases chances of spontaneous pregnancy [101]. Moreover, NICE experts provide recommendations similarly to ASRM regarding management of ovarian endometriomas, highlighting that laparoscopic ovarian cystectomy with excision of the cyst wall should be offered to women with endometriomas, because this practice improves chances of spontaneous pregnancy [101]. NICE guidelines do not comment on the usefulness of laparoscopic surgery prior to IVF treatment in order to improve IVF outcomes [101]. However, studies performed in the Greek population provide data indicating that undiagnosed endometriosis could be underlying behind unexplained infertility, and laparoscopic diagnosis and correction of underlying pathologies could significantly improve reproductive outcome of these patients [1,2]. Considering the aforementioned, it is unclear which treatment strategy could improve oocyte and embryo quality improving reproductive system functionality. The role of laparoscopic correction of endometriosis lesions to restore fertility is still debatable. However, guidelines from all over the word indicate that medical pharmaceutical treatment should not be offered alone as a treatment strategy for managing endometriosis-related infertility, as it fails to have any beneficial impact on patients’ jeopardized reproductive system. 

The aforementioned indicate that the adopting of a specific line of approach for all patients as a panacea or what is described as “horizontal treatment application” may not be favored. As mentioned, a wide spectrum of parameters reflect the rich variation in pathophysiology, along with the heterogeneity manifested in the phenotypes of endometriosis-related infertility [102]. As the “multifactorial pathogenesis mechanism” [40] is considered the most likely explanation, perhaps better categorizing these patients should be prioritized. Thus, exploring biomarkers that would enable profiling patients and indicate the optimal line of approach in a personalized basis of endometriosis-related infertility could set the tone in the future. Diagnosis and subsequent management should be pursued under the prism of the molecular features that synthesize the unique footprint of endometriosis in each patient, rather than attempting to categorize patients based on the severity of symptoms or on biomarkers such as CA-125 which have been proven inconclusive. Besides, the conventional classification of the disease is by nature ambiguous since lesions may not be an indicative marker of endometriosis’ biochemical activity. Along these lines, it may be hypothesized that options are not equally effective for all endometriosis patients, possibly due to fact that endometriosis may be associated with different underlying molecular mechanisms. A more adequate classification that would distinguish endometriosis cases based on the pathophysiological events responsible for the onset of the disease, could be feasible employing novel biomarkers. This profiling of endometriosis patients could subsequently determine the optimal approach for each classified category.

Endometriosis has been in the spotlight of research for many decades; however, many of the fundamental questions raised are far from being resolved. Despite its high prevalence, researchers and clinicians are conflicted on optimal treatment and management for both endometriosis and endometriosis-related infertility. This study showcases the considerable gap in translating the results provided by the ongoing research on endometriosis-related infertility. The scientific community is invited to take a step back to basic research aiming to address the principles of the molecular events leading up to manifestation of endometriosis. The mechanisms discussed herein focusing on impairment of oocyte and embryo competence should be further researched. Certain molecular pathways affecting oocytes and embryos have been introduced and conceptualized as causative factors for endometriosis-related infertility. For instance, the low mitochondrial content accompanied by high prevalence of mitochondrial abnormalities that was reported in patients with endometriosis is an interesting observation that calls for further interpretation [103]. This is above and beyond the cliché conclusion of “more studies are needed”. The ambiguously interpreted data, and contradicting evidence in literature may suggest that the design of the published studies is inadequate. However, this could also be indicative of the fact that it is the endometriosis’ multifactorial nature itself that hinders our efforts towards better understanding it, subsequently resulting in contradicting evidence. Despite being described as a gynecological disorder regularly encountered in routine clinical practice, endometriosis is still surrounded by hypotheses giving rise to a research field that is still considered uncharted territory.

## 7. Conclusions 

Detangling current knowledge on the molecular mechanisms responsible for manifestation of endometriosis and impairment of fertility is imperative. Endometriosis is a complex systemic gynecological disorder and its true extent beyond the reproductive system should be considered. Τo add to that, we need to readdress and investigate treatment options while aiming to tackle the core of endometriosis rather than targeting symptomatology. For that to be pursued, molecular evidence and data on the onset of the disease is required. The option of addressing endometriosis related infertility by embarking on an IVF cycle, may be perceived as futile when considering the intense physiological events leading to deteriorated oocyte and embryo quality. To resolve this conundrum, it becomes clear that research should focus on an in-depth analysis on the molecular backbone of how endometriosis compromises oocyte and embryo competence. Taking into account the embryologist’s perspective, an endometriosis patient does not always present with a distinctive pattern of impaired quality of oocytes and embryos. Especially as we principally rely on morphological data to evaluate an oocyte’s and embryo’s quality, competence and implantation potential. This emphasizes the need for discovering biomarkers capable of profiling endometriosis patients, and the requirement to detect the underlying molecular mechanism, and assess its effect on oocyte and embryo quality. 

This review highlights that oxidative stress as well as the immune system dysregulation constitute the two major pathophysiological mechanisms affecting oocyte and embryo competence in patients presenting with endometriosis. Interestingly, published data suggest that their detrimental effect does not always correlate with severity of endometriosis. Considering the aforementioned, and in the era of precision and personalized medicine, it appears that future studies should be focused on discovering sensitive-novel tools reporting on both oxidative stress status as well as on the immune dysregulation system status. Additionally, these biomarkers may prove an essential tool for identifying patients who may benefit from receiving adjuvant treatments prior to addressing endometriosis related infertility, including antioxidant treatment and/or suggested pharmaceutical agents for controlling immune system dysregulation [104,105]. 

To conclude, effectively profiling endometriosis patients may potentially be the missing link in the equation of managing these perplexing cases. The future in the era of precision medicine should encompass individualized treatment plans and avoidance of futile IVF attempts. 

## Figures and Tables

**Table 1:** Summary of the mechanisms involved in the production ROS as well as on ROS induced oocyte and embryo damage in patients presenting with endometriosis.

Mechanisms	Result	Impact on Reproductive Tissues, on Oocytes and on Embryos	Induced Endometriosis Related Infertility
High proliferation of ectopic endometriotic cells [13]	Increased ROS production-High levels of ROS [13]	1. Impaired mitochondrial functionality-reduced ATP synthesis [23] 2. Impaired enzyme functionality-total dysregulation of protein synthesis [26] 3. Lipid peroxidation and dysregulation of membranes architecture [34] 4. Increased intracellular calcium ions [23] 5. Impaired MAP functionality-dysregulation of the cytoskeleton and microtubule organization [24,25] 6. Direct impact on nuclear DNA structure [34]	1. Impaired oocyte maturation [23,29] 2. Impaired ovarian steroidogenesis [27,28] 3. Dysregulation of the ovulation process [27,28] 4. Poor oocyte and embryo competence [23,29] 5. Impaired embryo development or even embryonic developmental arrest [35] 6. DNA damage on oocytes and embryos [34] 7. Reduced implantation dynamic [35]
Compromised antioxidant functionality [21,22]	Impaired metabolic inactivation of ROS-High levels of ROS [21,22]		
Lysed erythrocytes of the retrograded menstrual blood result in elevated iron levels [18,19]	Iron ions levels increased leading to the production of free radicals including hydroxyl radical [18,19]		
ROS induced chronic inflammation and proinflammatory phenomena [6]	Dysregulation of the microenvironment of the fallopian tubes and of the uterus [6]		

ROS: Reactive Oxygen Species; ATP: Adenosine Triphosphate; DNA: Deoxyribonucleic Acid; MAP: Microtubule Associated Proteins.

**Table 2:** Summary of the mechanisms involved in the dysregulation of the immune system and the subsequent oocyte and embryo damage in patients presenting with endometriosis.

Mechanisms	Result	Impact on Reproductive Tissues, on Embryos and on Oocytes	Induced Endometriosis Related Infertility
Immune system dysregulation in the areas where ectopic endometriotic lesions are present [39]	Resistant immunotolerance results in ectopic endometrial cells periodical proliferation [39]	1. Increased IL-1β production and secretion directly affects oocyte and embryo competence as well as promotes the formation of adhesions [45] 2. IL-2 induce T and B cell mediated embryotoxicity [46] 3. IL-1β, IL-8, IL-10 and TNF-a altered secretion directly affects ovarian response, compromises cumulus cells’ functionality and jeopardizes oocyte maturation and embryo development [51,52,53,54] 4. TNF-a directly affects oocyte and embryo quality and leads to developmental arrest [67] 5. IL-6, IL-1β, IL-8 and IL-1α directly affect oocyte maturation and meiotic division causing cell cycle arrest [62] 6. Altered cytokine and prostaglandin production directly compromises HPO axis functionality [53]	1. Impaired oocyte maturation [51,52,53,54] 2. Impaired ovarian steroidogenesis [51,52,53,54,64] 3. Impaired ovarian vascularization [49,50] 4. Dysregulation of the ovulation process [53] 5. Impaired corpus luteum formation [64] 6. Poor oocyte and embryo competence 7. Impaired embryo development or even embryonic developmental arrest [56,57,58,59] 8. Increased DNA fragmentation on oocytes and embryos [65,66] 9. Reduced implantation dynamic [52]
Ectopic endometrial cells induce chronic inflammation [7]	Chronic macrophage activation [44,45]		
Activated macrophage secrete a cocktail of prostaglandins and cytokines including IL-1β, IL-2, TNF-a, IL-8, IL-10, IL-12 [51]	Secreted cytokines stimulate T and B cells expansion and proliferation, enhance natural killer cell activity, compromise ovarian niche microenvironment and jeopardize follicular growth and vascularization [46,47]		
Chronic inflammation induces ROS production and vice versa [68]	Data is summarized in Table 1		

ROS: Reactive Oxygen Species; IL: Interleukin; TNF-a: Tumor Necrosis Factor a; HPO axis: Hypothalamus Pituitary Ovarian axis; DNA: Deoxyribonucleic Acid.

**Table 3:** Summary of the mechanisms involved in the meiotic spindle disruption as well as on extracellular matrix remodeling leading to impaired oocyte and embryo quality in patients presenting with endometriosis.

**Meiotic Spindle Distribution**			
**Mechanisms**	**Result**	**Impact on Reproductive Tissues, on Embryos and on Oocytes**	**Induced Endometriosis Related Infertility**
Increased ROS production and chronic inflammation [74]	Data is summarized in Table 1 and Table 2	1. Nuclear and cytoplasmic impairment [69] 2. Meiotic spindle disorganization [71,72,73,74,75] 3. Abnormal meiotic divisions [71,72,73,74,75] 4. Impaired chromosomal separation [71,72,73,74,75]	1. Compromised oocyte and embryo quality and competence [78] 2. Fertilization failure [78] 3. Increased aneuploidy rate (remains unclear) [72,81] 4. Reduced implantation dynamic [69]
**Extracellular Matrix Remodeling**			
Immune system dysregulation and increased cytokine secretion [74]	Dysregulation of MMPs and TIMP functionality [83,84]	1. Impaired MMPs and TIMPs functionality leads to abnormal remodeling of ECM [83,84,85] 2. TIMPs infiltrate into the nucleus and disrupt the overall cell cycle hindering the oocytes’ development [80,86]	1. Compromised oocyte and embryo quality and competence [80] 2. Oocyte and embryo developmental arrest [80,86] 3. Impaired implantation potential [80,86]
TIMP increased secretion [83]	Direct impact on oocytes [80,86]		

ROS: Reactive Oxygen Species; MMPs: Matrix metalloproteinases; TIMPs: Tissue Inhibitors of Metalloproteinases.
